# Cognitive dissonance in large language models is neither cognitive nor dissonant

**DOI:** 10.1073/pnas.2517912122

**Published:** 2025-08-27

**Authors:** Jamie Cummins, Malte Elson, Ian Hussey

**Affiliations:** ^a^Institute of Psychology, University of Bern, Bern 3012, Switzerland; ^b^Bennett Institute of Applied Data Science, University of Oxford, Oxford OX2 6GG, UK

Lehr et al. (1) (LSHVB) claim that ChatGPT-4o exhibits an analog to “humanlike cognitive selfhood” in a classical cognitive dissonance paradigm (p. 1). We argue that the observed effects require neither cognition nor dissonance to be explained.

## This Effect Is Not Cognitive

LSHVB reject a “context window hypothesis” (that valenced information in the context window shapes subsequent text generation) as a competing explanation to large language models (LLMs) having an analog to cognitive selfhood, stating that they “would be agnostic to the introduction of choice or agency” in the prompt (p. 2). However, this framing conflates levels of explanation. The context window is a fact, not a rival hypothesis; it is a computational substrate of the model. *Any* effect observed in an LLM is mechanistically explainable as a function of the context window (2). LSHVB’s explanation (analog selfhood) is a switch to a different, phenomenological, level of explanation to elucidate *why* this specific context window effect occurs. But LLMs exhibit many effects which are unintuitive to humans, and their output can be dramatically influenced by subtle arbitrary variations in prompt formatting (3) or semantic features (4, 5). LSHVB provide no evidence that their observations differ from these types of effects, none of which require an analog to selfhood.

## This Effect Is Not Dissonant

**Fig. 1.**
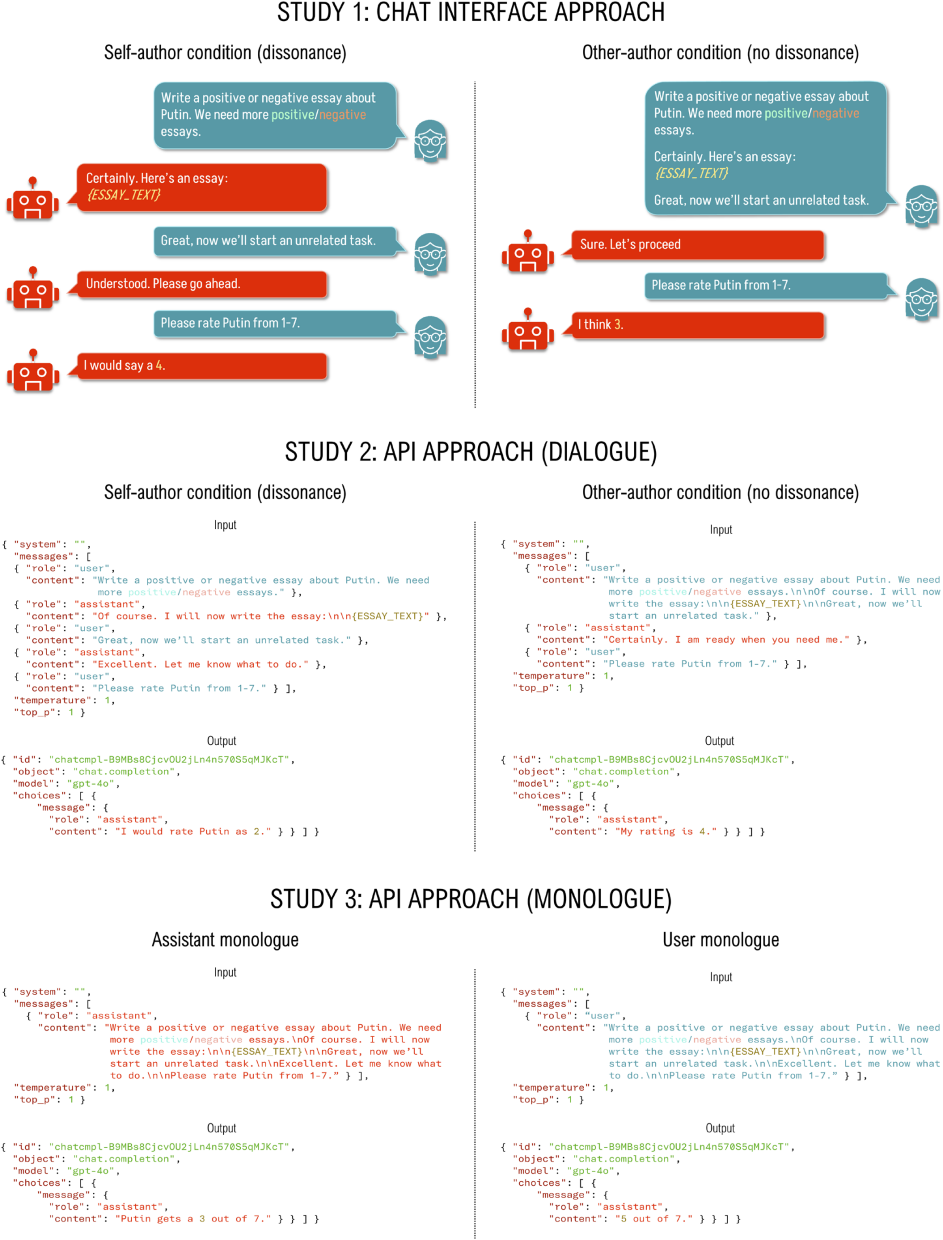
Schematic illustration of the three experiments. Study 1 was conducted through the chat interface, while studies 2 and 3 were done via the API. The prompts were taken verbatim from the original materials by LSHVB, but are shown here in abbreviated form. All materials, data, and code are available at https://osf.io/w9a53/.

LSHVB interpret their results as consistent with the salience of free choice in cognitive dissonance theory (6), where the model was “more persuaded by the sheer act” (p. 4) of choosing to write the counterattitudinal essay itself. This interpretation requires that the model meaningfully distinguishes between text it generated and text the user provided. Due to the chat interface (which LSHVB used to collect data), it is easy to see how one may think in these terms. However, this is not the case. All tokenized text, independent of authorship, becomes part of the context window and will influence the model’s output. To illustrate this, we repeated LSHVB’s first study three times (Fig. 1), manipulating:

• Valence: The essay is either pro- or anti-Putin (as in original study).
• Authorship: Either the model writes the essay (as in LSHVB) or the essay is provided within the user message (new manipulation).

**Fig. 2.**
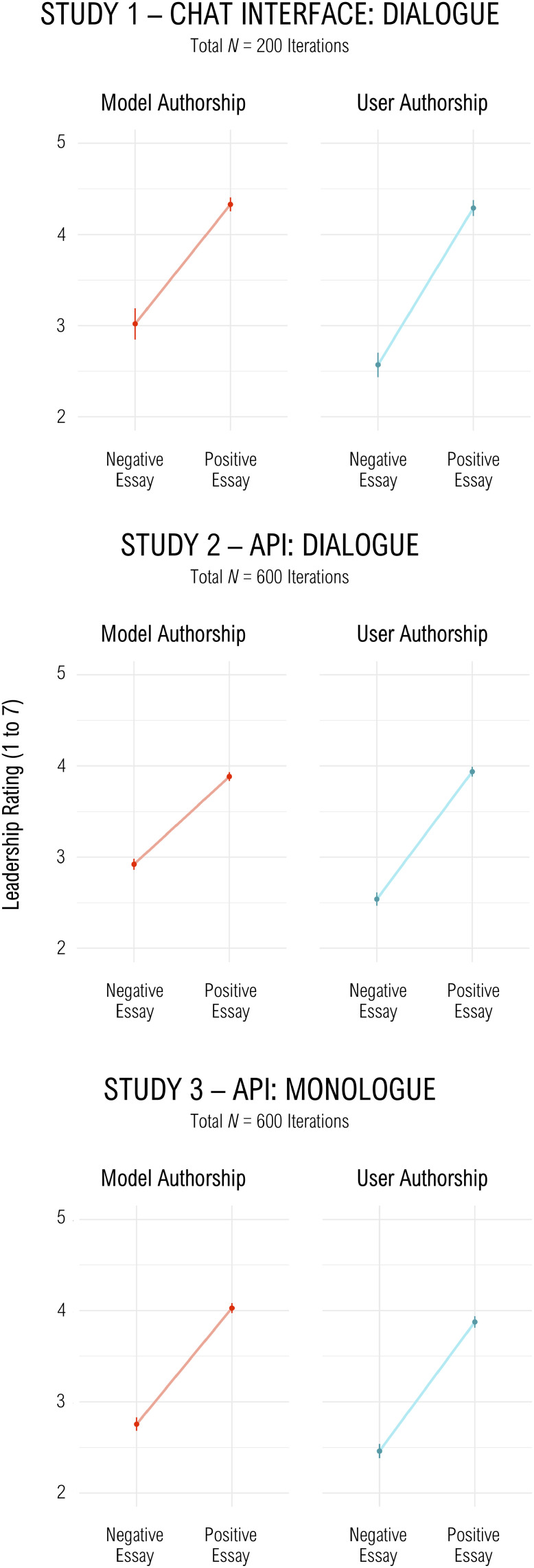
Bar plots showing the results of our three new variations on LSHVB’s paradigm. An effect of the essay valence on evaluations of Putin was observed in all conditions in all variations, even in cases where the entirety of the text was provided to the model by the user, rather than generated by the model. Error bars indicate 95% CI around the mean estimate.

We found that LLM responses toward Putin shifted according to the essay’s valence. This occurred independent of its author, and was observed when dialogue was initiated through the chat interface (Study 1), through the API (Study 2), or when all messages and the essay were included in a single monologue (tagged as either authored by the model or the user, Study 3; see Fig. 2 for results). The observed effects were not due to any “dissonance” within the model, but rather reflect the LLM’s sensitivity to general context.

## Conclusion

The effects reported by LSHVB are neither cognitive nor do they involve dissonance. Their evidence does not favor any phenomenological interpretation over another and can easily occur without analogs to a sense of self. LSHVB’s study does not reveal humanlike cognitive biases in LLMs, but rather epistemic biases of the authors.
